# Gut Microbiota’s Relationship with Liver Disease and Role in Hepatoprotection by Dietary Natural Products and Probiotics

**DOI:** 10.3390/nu10101457

**Published:** 2018-10-08

**Authors:** Xiao Meng, Sha Li, Ya Li, Ren-You Gan, Hua-Bin Li

**Affiliations:** 1 Guangdong Provincial Key Laboratory of Food, Nutrition and Health, Guangdong Engineering Technology Research Center of Nutrition Translation, Department of Nutrition, School of Public Health, Sun Yat-sen University, Guangzhou 510080, China; mengx7@mail2.sysu.edu.cn (X.M.); liya28@mail2.sysu.edu.cn (Y.L.); 2 School of Chinese Medicine, Li Ka Shing Faculty of Medicine, The University of Hong Kong, Hong Kong 999077, China; u3003781@connect.hku.hk; 3 Department of Food Science & Technology, School of Agriculture and Biology, Shanghai Jiao Tong University, Shanghai 200240, China; renyougan@sjtu.edu.cn; 4 South China Sea Bioresource Exploitation and Utilization Collaborative Innovation Center, Sun Yat-sen University, Guangzhou 510006, China

**Keywords:** gut microbiota, natural products, probiotics, hepatoprotection, mechanisms

## Abstract

A variety of dietary natural products have shown hepatoprotective effects. Increasing evidence has also demonstrated that gut microorganisms play an important role in the hepatoprotection contributed by natural products. Gut dysbiosis could increase permeability of the gut barrier, resulting in translocated bacteria and leaked gut-derived products, which can reach the liver through the portal vein and might lead to increased oxidative stress and inflammation, thereby threatening liver health. Targeting gut microbiota modulation represents a promising strategy for hepatoprotection. Many natural products could protect the liver from various injuries or mitigate hepatic disorders by reverting gut dysbiosis, improving intestinal permeability, altering the primary bile acid, and inhibiting hepatic fatty acid accumulation. The mechanisms underlying their beneficial effects also include reducing oxidative stress, suppressing inflammation, attenuating fibrosis, and decreasing apoptosis. This review discusses the hepatoprotective effects of dietary natural products via modulating the gut microbiota, mainly focusing on the mechanisms of action.

## 1. Introduction

Liver disease is a severe global health problem with high morbidity and mortality. Alcohol, drugs, toxicants, viruses, and obesity can cause liver disorders, including fatty liver, hepatitis, fibrosis, cirrhosis, and liver cancers [1,2,3]. The liver is in close association with the intestine, both anatomically and functionally, through the hepatic portal venous system. It has been reported that 10–100 trillion microorganisms from 300–500 different species estimated worldwide reside in the adult human gut [4,5]. The gut microbiota has been recently found to be involved in liver disease pathogenesis [6,7,8,9,10]. The composition of gut microbiota and their metabolites could play a key role in the cross-talk of the gut–liver axis, such as suppressing oxidative stress, inhibiting inflammation, and blocking hepatic lipid deposition [11,12,13,14]. Therefore, the gut microbiota might be a promising target to prevent and control liver diseases [15,16,17].

Many dietary natural products possess the abilities to protect against liver diseases [18,19,20]. They have shown the potential in improving the integrity of the gut mucosa, modulating the composition of the microbiota, reducing the toxic metabolites and translocated bacteria in the liver [21,22,23]. Some of the natural products can attenuate liver diseases by reverting gut dysbiosis, such as flaxseed oil, brown algae *Lessonia nigrescens*, and the herbal medicine *Qushi Huayu* decoction [23,24,25]. Some have also been observed to exert prebiotic effects, including oligofructose and epigallocatechin gallate (EGCG) in green tea [15,26]. In addition, natural beneficial bacteria like *Lactobacillus plantarum*, commonly found in fermented food products, have been illustrated to improve liver diseases as probiotics [27]. This review summarizes the role of the gut microbiota as a molecular target in the prevention and treatment of liver diseases using natural products, focusing on the mechanisms of action. In each part, the relationship between gut microbiota alteration and liver disease pathogenesis is firstly introduced, and then the mechanisms of how the intestinal microbiota affect liver diseases are discussed, followed by the beneficial effects and mechanisms of the natural products (including natural probiotics) on liver diseases by modulating gut microbe.

## 2. Non-Alcoholic Fatty Liver Disease (NAFLD)

Non-alcoholic fatty liver disease (NAFLD) is epidemic worldwide, particularly in Western countries, and more than one-fourth of the US population suffer from NAFLD [28]. It is often accompanied with obesity and type 2 diabetes. An association between NAFLD and a higher risk of liver cancer has also been found [29,30]. It was reported that 11% of hepatocellular carcinoma (HCC) cases were attributable to NAFLD in the USA and 22% in Germany. The pathogenesis of NAFLD is intensively associated with lipid accumulation, oxidative stress, and inflammation. Certain natural products have shown promising beneficial effects on liver pathological progresses [31,32,33].

Overwhelming evidence indicates that the gut microbiota is closely related to the pathogenesis of NAFLD [34,35,36]. Gut microbiota could be an independent contributor to the development of NAFLD in the face of obesity, as it was the distinctive differences of the gut microbiota at the levels of phylum, genera, and species that determined the response to a high-fat diet (HFD) in mice [37]. It has been revealed that the abundance of 5 *Lactobacillus* spp. (*L. zeae*, *L. vaginalis*, *L. brevis*, *L. ruminis* and *L. mucosae*) increased in children with NAFL/non-alcoholic steatohepatitis (NASH)/obesity, while the abundance of 3 *Bifidobacterium* spp. (*Bifidobacterium longum*, *Bifidobacterium bifidum* and *Bifidobacterium adolescentis*) decreased [38]. In addition, NAFLD patients were found with a significant over-representation of *Lactobacillus* species and some phylum Firmicutes (Lachnospiraceae; genera *Dorea*, *Robinsoniella*, and *Roseburia*), as well as a significant under-representation in phylum Firmicutes (Ruminococcaceae; genus *Oscillibacter*). Also, a significant elevation of volatile organic compounds (VOC) was observed in their feces [8].

Gut microbiota participated in the progression from NAFLD to HCC, and its altered profile might indicate the stages and features of NAFLD development [39,40]. For instance, NAFLD onset was characterized with a decrease of *Oscillospira*, and progression of NAFLD with increased *Ruminococcus* and *Dorea* [39]. It was also reported that the dramatically increased amount of DNA from *Lactobacillus gasseri* and/or *Lactobacillus taiwanensis* possibly contributed to the pathogenesis of steatohepatitis in mice [41]. Moreover, an increased percentage of Gram-negative bacteria compared to that of Gram-positive bacteria, an increased Gram-negative Proteobacteria, and a reduced ratio of Bacteroidetes over Firmicutes, were considered to play a role in promoting liver fibrogenesis in NAFLD [42]. Additionally, NAFLD could be deteriorated because of lipopolysaccharide (LPS) [43] or due to a gut flora perturbance, such as topological shifts and growth promotion [44]. In a mouse model of non-alcoholic steatohepatitis-HCC (NASH-HCC), the markedly increased bacterial species, such as *Atopobium* spp., *Bacteroides* spp., and *Desulfovibrio* spp., showed a positive correlation with altered LPS levels and pathophysiological observations [45]. Additionally, gut microbiota alteration could independently indicate the severity of NAFLD, namely, Streptococcaceae [46] and *Bacteroides* increase for NASH, and *Ruminococcus* increase for significant fibrosis, providing predictive effects on chronic liver diseases as well as the possible targets of treatment [47]. Furthermore, a positive association was found between small intestinal bacterial overgrowth and the frequency of NAFLD [48].

The mechanisms by which intestinal microbiota influence NAFLD have been investigated. Evidence shows that the gut microbiota dysbiosis, inflammation, and the impaired mucosal immune function were possible contributors in the development of NAFLD [49,50]. Besides the consequent bacterial translocation, leaked relevant products, such as LPS and VOC, derived from the gut microbiota entered the liver through the portal venous circulation and promoted the pathogenesis of NAFLD [43,51]. Moreover, much higher endogenous alcohol production, as a constant oxidative stressor, together with an increase in energy production with reduced carbohydrate and amino acid metabolism, possibly contributed to the progression of NAFLD [52]. In addition, the gut flora was evidenced to influence the pathogenesis of NAFLD by alternating bile acids [53,54].

Studies have shown protective effects of natural probiotics against NAFLD (Table 1). For instance, *Lactobacillus johnsonii* BS15 effectively prevented NAFLD in mice by enhancing the antioxidant defense system, suppressing insulin resistance, restoring mitochondrial functions, improving intestinal permeability, and modulating the gut flora [55]. In another study, treatment with *Lactobacillus rhamnosus* GG, 5 × 10^7^ colony-forming units (CFU)/g, protected against high-fructose diet-induced NAFLD in mice by altering the beneficial bacteria in the distal small intestine like butyrate-producing Firmicutes, improving the intestinal barrier, reducing LPS levels in portal venous blood, attenuating inflammation, and inhibiting fatty acid accumulation in the liver [56]. Additionally, a combination of probiotics (0.5 × 10^6^ CFU of live *Bifidobacterium infantis* and *Lactobacillus acidophilus* plus 0.5 × 10^5^ CFU live *Bacillus cereus*) was found to inhibit the progression of NAFLD in rats fed with high sucrose and an HFD [57]. Notably, such a probiotic supplementation showed the ability of ameliorating gut microbiota dysbiosis, restoring intestinal barrier integrity, decreasing serum inflammatory cytokines, attenuating elevated serum liver enzymes and glycometabolic biomarkers, and improving liver pathology, possibly through the LPS/toll-like receptor 4 (TLR4) signaling pathway, as TLR4 could recognize LPS and then activate immune cell responses. Moreover, a synbiotic comprising *Lactobacillus fermentum* CECT5716 and fructo-oligosaccharides was demonstrated to prevent hepatic steatosis and mitigate insulin resistance in high fructose-fed rats, and the underlying mechanisms likely included the modulation of gut microbiota, accompanying markedly improved dysbiosis and barrier function [58].

Of note, certain natural products and their bioactive compounds have been demonstrated to alleviate NAFLD (Table 1). The extracts of 5 arctic berries, namely *Vaccinium uliginosum* L., *Empetrum nigrum* L., *Rubus chamaemorus* L., *Arctostaphylos alpina* L., and *Vaccinium vitis-idaea* L., were tested in mice fed a high-fat/high-sucrose diet, the last 3 of which showed the capabilities of attenuating hepatic steatosis, reducing circulating endotoxins, decreasing inflammation in the gut and intestine by targeting the gut–liver axis, featured by an increased presence of *Akkermansia muciniphila*, *Turicibacter* and *Oscillibacter* [59]. Perilla oil and fish oil have also been investigated, and both of them alleviated NAFLD as they could slightly restore the decreased relative abundance of Gram-positive bacteria in the gut caused by an HFD and counteract the increased abundance of *Prevotella* and *Escherichia* [60,64]. In particular, fish oil increased the relative abundance of *Akkermansia*, which were thought to ameliorate obesity. Moreover, the hepatoprotective effects of phytic acid were shown to reduce upregulated expression of hepatic lipogenic enzymes and modulate intestinal microbiota by increasing *Lactobacillus* spp., decreasing *Clostridium cocoides*, and inhibiting elevated *Clostridium leptum* caused by a high-sucrose diet [21]. Additionally, citrulline has been reported to ameliorate hypertriglyceridemia and steatosis, and mitigate Western diet-induced liver injuries in rats by restricting lipid deposition, enhancing insulin sensitivity, suppressing inflammation, and restoring antioxidant status, which were partially attributed to the improvement of intestinal barrier function and the altered gut microbiota [61,62].

The herbal medicine *Qushi Huayu* decoction and its active components (geniposide and chlorogenic acid) have shown protective effects on NAFLD by decreasing serum LPS possibly because it reduced the colonic mucosal damage, promoted the regulatory T cell (Treg)-inducing bacteria, and down-regulated inflammation, leading to restored gut barrier function and reduced hepatic exposure to microbial metabolites [23]. Moreover, red pitaya β-cyanins caused a distinct increase of relative abundance of *Akkermansia* as well as a decreased ratio of *Firmicutes* and *Bacteroidetes* in HFD-fed mice, and also improved inflammatory profile, suggesting its clinical potential in managing NAFLD and obesity [22]. In addition, quercetin was suggested to be used as a novel therapeutic option for NAFLD due to its prebiotic capacity, which could improve gut dysbiosis by inhibiting endotoxemia-mediated TLR4/NF-κB signaling pathway activation, suppressing the subsequent inflammation and induced reticulum stress, and blocking the deregulation of lipid metabolism gene expression [16]. Furthermore, an analog of resveratrol, 2,3,5,4′-tetrahydroxy-stilbene-2-*O*-*β*-*D*-glucoside (TSG), was investigated in rats with HFD-caused NAFLD [63]. The results showed that TSG could prevent NAFLD by balancing gut flora, improving integrity of intestinal mucosal barrier, and decreasing serum LPS levels via TLR4/NF-κB pathway.

**Figure 1:**
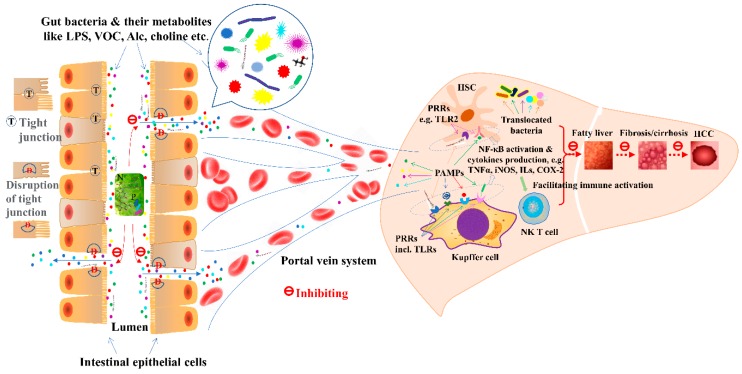
The inhibitory effects of natural products on the cascade of microbial translocation in liver disease progression. *Abbr.*: Alc, alcohol; D, disruption of tight junctions; HCC, hepatocellular carcinoma; HSC, hepatic stellate cell; ILs, interleukins; incl., including; LPS, lipopolysaccharide; N, natural products and bioactive components; PAMPs, pathogen-associated molecular patterns; PRRs, pattern recognition receptors; T, tight junction; TLRs, toll-like receptors; Treg, the regulatory T cell; VOC, volatile organic compounds.

Taken together, increasing evidence has shown that NAFLD onset, development, and severity as well as the incidence rate, are closely associated with the gut microbiota. Various natural products have been demonstrated to protect liver and restore its function by altering gut flora composition, enhancing integrity of the gut barrier, reducing gut-derived endotoxin, suppressing inflammation, and restricting oxidative stress. Notably, LPS/TLR4/NF-κB pathway is commonly involved (Figure 1).

## 3. Alcoholic Liver Disease (ALD)

Alcohol overconsumption is linked to many diseases, contributing to around 6% of all deaths worldwide [3,65]. Long-term alcohol overconsumption or binge drinking could result in a spectrum of disorders including alcoholic liver disease (ALD) [66]. Some natural products have been used as liver protective agents since ancient times across cultures. Increasing research has been recently conducted on their effects and mechanisms against liver diseases caused by alcohol overconsumption [67,68].

Intestinal dysbiosis often occurs after alcohol exposure. Some bacteria, such as *Akkermansia muciniphila*, *Porphyromonadaceae* and *Parasutterella*, were demonstrated to decrease in the setting of ALD, while some increased including *Firmicutes* and *Parabacteroides* [24,69]. Such unbalanced microbiota often cause or aggravate ALD. Alcohol consumption, especially binge drinking, could cause the enterocytes apoptosis as well as degradation of intestinal tight junction and adherens junction proteins, contributing to the gut leakiness endotoxemia, inducing hepatic inflammation or exacerbating liver diseases [70]. In rats with ALD, the gut epithelial permeability increased, more endotoxins and other bacterial metabolites were released into portal circulation, causing more severe liver inflammation and injury, possibly resulting from more phosphorylation of Forkhead box ‘Other’ (FoxO) proteins induced by elevated tumor necrosis factor α (TNFα) [71]. Moreover, not only did intestinal microbiota play a crucial role in the development of ALD, but it was one of the major contributors to the individual susceptibility to ALD as well [72].

A variety of probiotics have shown beneficial effects on ALD via gut flora alteration (Table 2). For instance, *Lactobacillus rhamnosus* GG was evidenced to reduce ALD through suppressing hepatic inflammation and counteracting the increased mRNA expression of TLRs and CYP2E1, and the phosphorylation of p38 MAP kinase induced by alcohol [73]. *Lactobacillus rhamnosus* GG could also ameliorate alcoholic fatty liver in mice by decreasing fatty acids in the liver, strengthening intestinal barrier and reducing endotoxemia [74]. In addition, *Lactobacillus rhamnosus* R0011, *acidophilus* R0052, Korea red ginseng, and urushiol from *Rhus verniciflua* Stokes were reported to ameliorate ALD in mice by reducing the inflammatory mediators such as TNFα, IL-6, and IL-10, and downregulating TLR4 expression [75]. Another example is *Akkermansia muciniphila*, which attenuated hepatic injury as well as steatosis and reduced neutrophil infiltration due to its protective effects of increasing mucus production, which resulted in the enhanced intestinal barrier integrity [69].

**Figure 2:**
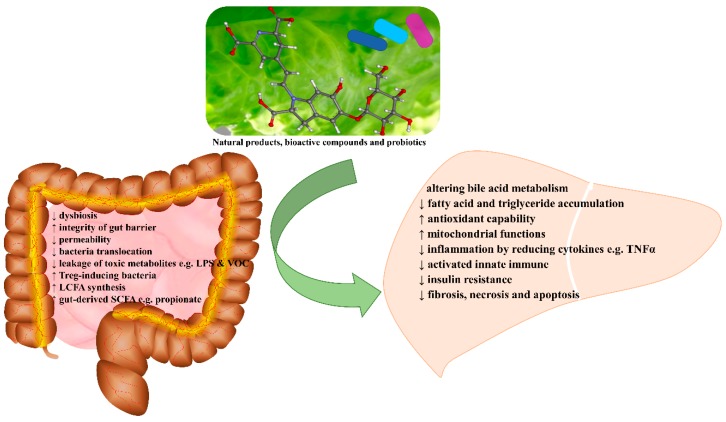
Hepatoprotective effects and mechanisms of natural products via gut microbiota.

Many natural products were demonstrated beneficial impacts on ALD and the gut microbiota has been found participating in the mechanisms of hepatoprotection (Figure 1 and Figure 2) [76,77,78]. Flaxseed oil, for instance, was observed to relieve ALD in mice by reducing inflammatory cytokines and modulating gut dysbiosis [24]. Additionally, the relationship between gut flora, dietary lipids and the pathogenesis of ALD was investigated [79]. Saturated long chain fatty acid (LCFA) supplementation could promote commensal *Lactobacillus growth*, maintain intestinal eubiosis and the gut barrier, so as to ameliorate ALD, as alcohol reduced proportion of *Lactobacillus* species and blunted the capability of biosynthesizing LCFA. The hepatoprotective effects were also observed in mice with ALD fed lychee (*Litchi chinensis* Sonn.) pulp phenolic extract, presenting alleviated intestinal microbiota dysbiosis, restored intestinal barrier dysfunction, and suppressed liver inflammation [80]. It was also reported that green tea extract could prevent NASH by enhancing the tight junction proteins, decreasing endotoxin leak, and suppressing TLR4/MyD88/NF-κB activation [81]. Rhubarb extract protected the liver from inflammation and oxidative stress induced by binge alcohol consumption in a mouse model, and such effects were illustrated to be associated with the modulation of the gut microbiota such as increasing *Akkermansia muciniphila* and *Parabacteroides goldsteinii* [82]. In addition, milk osteopontin was demonstrated to protect the intestine and the liver in mice fed with alcohol, as it could maintain intestinal integrity and permeability due to the preserved expression of tight-junction proteins, showing the normalized inflammation-related biomarkers and cytokines in both liver and plasma, as well as the improved parameter profile associated with endotoxemia [83]. Another naturally existing compound, indole-3-carbinol, found in cruciferous vegetables, has shown hepatoprotective effects on ALD by reducing oxidative stress and inflammation related to the gut-liver-adipose tissue axis [84]. Additionally, it inhibited apoptosis of enterocytes and modulated tight junction protein claudin-1, leading to improved gut integrity and endotoxemia; it also restored the antioxidant capacity of the liver and blocked the release of free fatty acids. Furthermore, aplysin from the red alga *Laurencia tristicha* was reported to revert the increased plasma endotoxin diamine oxidase (DAO) and fatty acid-binding protein 2 (FABP2) as well as the altered gut microbial composition in rats with ALD [85]. In another study, when administrated in a double layered microencapsulation, *L. plantarum* was shown to attenuate endotoxemia, normalize transaminases, reduce NF-κB and other cytokines, and restore the morphology and function of the gut and liver [27]. Moreover, EGCG exhibited a strong prebiotic effect on *L. plantarum* [26]. Meanwhile, better hepatoprotective effects were observed with the administration of a synbox microencapsulated both EGCG and *L. plantarum* than simultaneously-administrated free agents, showing decreased levels of blood alcohol and endotoxins as well as the improved liver function via anti-inflammation, anti-necrosis, and anti-apoptosis.

Collectively, intestinal dysbiosis is tightly associated with the pathogenesis of ALD, against which various natural products (e.g., flaxseed oil, lychee, and green tea) and probiotics (e.g., *Lactobacillus rhamnosus* GG) have been proven effective (Table 2). Many of the tested products or probiotics showed the potential to be used as novel therapeutic strategies for ALD by modulating gut microbiota.

## 4. Liver Fibrosis and Cirrhosis

Liver injuries often develop into fibrosis and even cirrhosis with collagen-rich extracellular matrix accumulation and scar tissue hyperplasia, speeding up the pathological progress of liver diseases and resulting in HCC, with many cells involved such as hepatic stellate cells (HSCs), portal myofibroblasts, and bone marrow derived cells [86].

Accumulative studies have shown that the gut-liver axis participates the liver fibrotic progression [87]. It was observed in a cholestatic mouse model that TLR2 was associated with intestinal barrier function, and TNF receptor I is one of the key mediators of signals on intestinal epithelial cells to facilitate the bacterial translocation, so as to accelerate the progression of liver fibrosis [88]. Moreover, it has been clarified that the pathogen-associated molecular patterns (PAMPs) translocated from a leaky gut, entered the liver through the portal vain, and then activated several TLRs in hepatic macrophages, leading to the interferon β (IFNβ) upregulation and the alterations of antibacterial cytokines [89]. The impacts of IFNβ overexpression could be amplified by intracellular bacterial infection in the liver. In addition, gut microbial translocation in fibrotic mouse liver could lead to the hepatic tonic type I interferon expression which, together with bacteria-caused intracellular infection, could induce the IFN and IL-10 production from myeloid cells [90]. Consequently, cytosolic pattern recognition receptors (PRRs) were activated. In short, the microbial translocation triggered a cascade, resulting in immune dysfunction and antibacterial defense failure, even infection-associated mortality. Furthermore, gut-derived products in the liver via the portal vein could induce lipocalin-2, which is derived from the spleen and involved in Kupffer cell regulation, resulting in retarded development of liver fibrosis [91].

The aforementioned mechanisms indicated that the development of liver fibrosis was interactive with the gut microorganisms. Therefore, inhibitors of interleukins or interferon, and the agents that improve the gut barrier could benefit liver fibrosis. It was reported that the oral administration of *Saccharomyces boulardii* improved the gut permeability, normalized the increased serum endotoxin and pro-inflammatory cytokine levels, and modulated intestinal microbial composition in rats with CCl_4_-induced liver fibrosis [92].

In advanced liver diseases like liver cirrhosis with or without complications, gut flora alterations have been reported [93]. Quantitative metagenomics results showed that 75,245 genes were different in abundance between cirrhotic patients and healthy individuals, which could be classified into 66 clusters standing for cognate bacterial species, among which 28 clusters are enriched in patients [7]. In addition, significant increase of Enterobacteriaceae and Enterococcus, and decreased ratio of *Bifidobacterium* genus and Enterobacteriaceae were observed in cirrhotic patients, and Eubacteria and the Bacteroides-Prevotella group were negatively associated with plasma endotoxin and IL-6 as well as fecal secretory IgA, which significantly increased in cirrhotic patients [94,95]. Furthermore, the counts of *Lactobacilli* enriched in the stool samples of cirrhotic patients, including hydrogen peroxide-producing strains [96]. In patients with alcoholic liver cirrhosis, Enterobacteriaceae increased, while some obligate anerobic bacteria significantly decreased [97]. In addition, it was illustrated by a median 27 times more Enterobactericeae DNA was found in feces of alcoholic cirrhotic patients than healthy subjects, and Enterobactericeae was the most common bacteria translocating into the liver of the patients [98]. Similarly, multiple shifts in the intestinal microbiota were found in another group of alcoholic cirrhotic patients, including enrichment in Bifidobacterium and Lactobacillus, as well as the increased tendency of toxin synthesis like acetaldehyde, which was associated with the colorectal cancer and other pathologies [99]. In addition, gut flora changes with cirrhotic progression. For instance, the relative abundance of Lactobacillaceae and Enterococcaceae was observed higher in cirrhotic patients without hepatic encephalopathy (HE) than that in healthy participants; whereas, the highest relative abundances were found in the cirrhotic patients with HE [100]. It was also illustrated that endotoxemia was getting worse as the liver cirrhosis got advanced [101]. Another example was that endotoxemia was reduced and cognition was improved after rifaximin treatment in cirrhotic patients with minimal HE, indicating intestinal bacteria modulation might be effective against cirrhosis [102].

Probiotics and natural products have been demonstrated to promote gut flora restoring and benefit patients with cirrhosis (Table 3). For example, the decreased levels of rapid-turnover proteins in the serum of patients with alcoholic liver cirrhosis were reverted by the treatment of Yakult 400, a probiotic beverage containing *Lactobacillus casei* strain Shirota [97]. Furthermore, a clinical trial of probiotic VSL#3 (daily for 6 months) was carried out in Indian patients with HE, and the result showed that probiotic VSL#3 reduced the severity of liver cirrhosis and hospitalization [103] In another study, VSL#3 was administrated to some Polish patients with liver cirrhosis in a short period (daily for 28 days) [104]. The results suggested that several molecules were modulated, such as macrophage inflammatory protein 3α (MIP-3α)/chemokine ligand 20 (CCL20), nitric oxide (NO), thromboxane 2 (TXB2) and myeloperoxidase (MPO), indicating the therapeutic potential of probiotics on liver cirrhosis. Recently, administration of *Lactobacillus salivarius* LI01 or *Pediococcus pentosaceus* LI05 was reported to improve the disrupted intestinal barrier in rats with CCl_4_-induced liver cirrhosis, presenting decreased pathogenic bacteria (e.g., *Escherichia*) and increased potential beneficial bacteria (e.g., *Elusimicrobium* and *Prevotella*) [105]. Moreover, after treated with either of the two mentioned probiotics, profibrogenic genes in the liver were downregulated, serum endotoxins and bacterial translocations were reduced, intestinal mucosal ultrastructure was improved, and hepatic inflammatory cytokines and TLRs were decreased. Additionally, artesunate supplementation could attenuate liver cirrhosis by improving gut microbial dysbiosis, suppressing inflammation, enhancing the intestinal mucosal barrier, and reducing bacterial translocation [106] (Figure 2).

In summary, intestinal dysbiosis has a close relation with the outcomes of liver fibrosis and cirrhosis. Evidence showed that probiotics like *Saccharomyces boulardii* and *Lactobacillus casei* strain Shirota, and certain natural products like artesunate could help to attenuate fibrosis and/or cirrhosis by maintaining intestinal eubiosis, increasing integrity of the gut barrier, decreasing translocation of the bacteria and their metabolites, ameliorating endotoxemia, and reducing inflammation with TLRs involved (Table 3) (Figure 2).

## 5. Liver Cancer

As one of the leading causes of death, cancer resulted in 8.8 million deaths worldwide in 2015, and in particular, 0.8 million was caused by liver cancer [2,108]. Growing attention has been paid to the anticancer effects of natural products and their bioactive compounds such as polyphenols, which possess potent antioxidant, anti-inflammatory and immune-modulating activities [13,109,110,111]. Therefore, they might be used to prevent or treat cancers directly or as adjuvants to enhance the performance and/or reduce side effects of anticancer therapies [112,113,114].

Increasing evidence has shown that intestinal dysbiosis plays a crucial role in the progression of liver cancer, and some altered relative abundance of bacteria might be the potential risk indexes and therapeutic targets for HCC [115]. It was found that fecal counts of *Escherichia coli* were significantly increased in patients with HCC than those in patients with cirrhosis, indicating hepatocarcinogenesis may attribute, at least partially, to the *E. coli* overgrowth [116]. It was also pointed out that the development of HCC in obese mice was accelerated by deoxycholic acid (DCA), a gut bacterial metabolite, by provoking senescence-associated secretory phenotype (SASP) in HSCs [6,117]. DCA was increased due to obesity-induced gut flora alteration, mainly the increased *Clostridium* cluster, and it could cause DNA damage. Moreover, the senescent HSCs produced cytokines such as IL-1β and caspase-1, promoting HCC progression in chemical carcinogen-exposed mice. Besides synergy with DCA, lipoteichoic acid, a gut microbiota component, was found to translocate into liver, upregulate SASP and COX-2 in the senescent HSCs via TLR2. Furthermore, COX2 overexpression gave rise to prostaglandin E_2_ (PGE_2_) generation, which contributed to HCC progression by inhibiting the antitumor immunity via PGE_2_ receptor 4 [118]. In addition, intestinal disintegrity and increasing bacterial translocation were associated with HCC recurrence after liver transplantation and resection through the LPS/TLR4 pathway, which could be attenuated by modulating the gut–liver axis, gut sterilization, and TLR4 antagonism [119]. Furthermore, altered gut microbiota resulted in the disorders of bile acid synthesis and reabsorption, and the persistent retention of high-concentration bile acids in the liver. The excessive hepatic bile acids are carcinogenetic in the presence of obesity by affecting hepatocyte metabolism, enhancing oxidative stress, promoting the production of pro-inflammatory cytokines such as TNFα, and IL-1β from Kupffer cells, and increasing LPS production and absorption [120]. Therefore, modulating bile acid conversion might be a novel strategy for liver cancer management. Interestingly, gut microbiome showed an inhibitory effect on both primary and metastatic liver tumors by increasing hepatic CXCR6^+^ natural killer T (NKT) cells via modulating bile acid conversion [121].

Inulin-type fructans from natural products have been used as a prebiotic to reduce cancer cell proliferation in mice transplanted with hepatic BaF3 cells by promoting gut microbiota to produce short-chain fatty acids (SCFA) like propionate [107] (Table 3). The mechanisms of such protective effects were considered in relation to gut microbiota modulation and increase of propionate in the portal vein. In the same study, the in vitro test results showed that SCFA suppressed BaF3 cell proliferation, and specifically, propionate inhibited BaF3 cell growth in a cAMP-dependent manner. In addition, EGCG metabolites were thought to exhibit anticancer effects, with the gut microbiota-mediated metabolism as the necessary prerequisite [17].

To summarize, gut flora and their metabolites are actually linked to the progression of liver cancer, which could be indicated by specific alterations such as *E. coli* overgrowth, exacerbated by DCA, while inhibited by propionate. Natural products, like inulin-type fructans, exhibited antiproliferative impacts on liver cancer by modulating gut flora and promoting gut-derived SCFA production.

## 6. Conclusions

The relationship between gut microbiota and liver diseases has been intensively investigated. A variety of liver diseases are characterized with changes of intestinal flora, and some of the altered species were considered to predict the outcomes of liver diseases, such as *Oscillospira* decrease for the onset of NAFLD and *Bacteroides* increase for the severity of NASH, indicating the potential therapeutic targets. Meanwhile, gut dysbiosis led to poor integrity of the gut barrier and increased the leak of translocated bacteria and toxic metabolites, which could reach the liver via the portal vein system. The consequent disturbance of bile acid metabolism and hepatic lipid accumulation, together with oxidative stress and increased inflammation accelerated liver disease progression through LPS/TLRs/NF-κB signaling pathways. Collectively, changes of the gut microbiota and their metabolites can affect pathogenesis of liver diseases and vice versa. Some probiotics have shown hepatoprotective effects including *Lactobacillus rhamnosus* GG and *Akkermansia muciniphila*. Natural products (e.g., oligofructose and quercetin) have been illustrated to ameliorate liver diseases by modulating gut flora, improving intestinal permeability, and altering the primary bile acid. Consequently, the pathogenic progression of liver diseases has been reverted to some degree, showing reduced hepatic lipid accumulation, relieved endotoxemia, inhibited oxidative stress, suppressed inflammation, attenuated fibrosis, and decreased apoptosis and necrosis. Therefore, those products targeting intestinal microbiota modulation might be cost-efficient and effective therapeutics for the prevention and treatment of liver diseases. Given the promising application in functional foods and pharmaceuticals, it is worth detecting more natural products as prebiotics or probiotics with hepatoprotective effects in future. The effective components of natural products and their synergists/antagonists need to be qualified and quantified. Their features regarding application such as bioavailability, effective dose, and side effects are also worth investigating. More attention should also be paid to illustrating the mechanisms of action, providing more targets to manage liver diseases. Clinical trials are also warranted to provide practical and economical strategies to manage liver diseases using natural products by modulating the gut microbiota.

## Figures and Tables

**Table 1:** Effects and mechanisms of natural products and probiotics on non-alcoholic fatty liver disease (NAFLD) by modulating gut microbiota.

Factors that Affect NAFLD	Study Type	Effects and Mechanisms	References
**Probiotics**			
*Lactobacillus johnsonii* BS15 (2 × 10^7^ colony-forming units (CFU)/0.2 mL or 2 × 10^8^ CFU/0.2 mL)	In vivo (in mice)	Enhancing antioxidant defense system, suppressing insulin resistance, restoring mitochondrial functions, improving intestinal permeability, and modulating gut flora	[55]
*Lactobacillus rhamnosus* GG (5 × 10^7^ CFU/g body weight)	In vivo (in mice)	Altering the beneficial bacteria in the distal small intestine, improving the intestinal barrier, reducing lipopolysaccharide (LPS) levels in portal venous blood, attenuating inflammation, and inhibiting fatty acid accumulation in the liver	[56]
A combination of live *Bifidobacterium infantis* and *Lactobacillus acidopilus* (0.5 × 10^6^ CFU) and live *Bacillus cereus* (0.5 × 10^5^ CFU)	In vivo (in rats)	Ameliorating gut microbiota dysbiosis, restoring intestinal barrier integrity, decreasing serum inflammatory cytokines, improving liver pathology, attenuating increased serum liver enzymes and glycometabolic biomarkers, possibly through the LPS/toll-like receptor 4 (TLR4) signaling pathway	[57]
A synbiotic comprising *Lactobacillus fermentum* CECT5716 and fructo-oligosaccharides	In vivo (in rats)	Preventing hepatic steatosis and mitigating insulin resistance through modulation of gut microbiota, accompanying markedly improved dysbiosis and barrier function.	[58]
**Natural products**			
Arctic berries extract (*Vaccinium uliginosum* L., *Empetrum nigrum* L., *Rubus chamaemorus* L., *Arctostaphylos alpina* L., and *Vaccinium vitis-idaea* L.)	In vivo (in mice)	(last 3 berries) Attenuating hepatic steatosis, reducing circulating endotoxins, decreasing inflammation in the gut and intestine by targeting the gut–liver axis, featured by an increased presence of *Akkermansia muciniphila*, *Turicibacter* and *Oscillibacter*	[59]
Perilla oil and fish oil	In vivo (in rats)	Both: slightly restoring the decreased relative abundance of Gram-positive bacteria in the gut and counteracting the increased abundance of *Prevotella* and *Escherichia*. Fish oil: increasing the relative abundance of *Akkermansia*	[60]
Phytic acid	In vivo (in rats)	Reducing upregulated expression of hepatic lipogenic enzymes induced by a high-sucrose diet, and modulating intestinal microflora	[21]
Citrulline	In vivo (in rats)	Modulating gut microbiota, resulting in restricted lipid deposition, enhanced insulin sensitivity, suppressed inflammation, and restored antioxidant status	[61,62]
Herbal medicine *Qushi Huayu* decoction	In vivo (in rats)	Decreasing serum LPS possibly by reducing colonic mucosal damage, promoting Treg-inducing bacteria, and down-regulating inflammation	[23]
Red pitaya β-cyanins	In vivo (in mice)	Markedly altering intestinal flora like increasing the relative abundance of *Akkermansia* as well as decreasing the ratio of *Firmicutes* and *Bacteroidetes*, and improving inflammatory profile	[22]
Quercetin	In vivo (in mice)	Reverting gut dysbiosis, inhibiting activated TLR4/NF-κB signaling pathway, suppressing the subsequent inflammation and induced reticulum stress, and blocking the deregulation of lipid metabolism gene expression	[16]
TSG (2,3,5,4′-tetrahydroxy-stilbene-2-*O*-*β*-*D*-glucoside)	In vivo (in rats)	Balancing gut flora, improving integrity of intestinal mucosal barrier, and decreasing serum LPS levels through TLR4/NF-κB pathway	[63]

**Table 2:** Effects and mechanisms of natural products and probiotics on alcoholic liver disease (ALD) by modulating gut microbiota.

Factors that Affect ALD	Study Type	Effects and Mechanisms	References
**Probiotics**			
*Lactobacillus rhamnosus* GG	In vivo (in mice); in vitro (human peripheral blood monocytes-derived macrophages)	Suppressing tumor necrosis factor α (TNFα) production and inflammation, counteracting the increased mRNA expression of TLRs and CYP2E1, and phosphorylation of p38 MAP kinase; dose-dependently reducing TNFα, decreasing hepatic fatty acids, enhancing long chain fatty acid (LCFA) synthesis, strengthening intestinal barrier, and reducing endotoxemia	[73]
*Lactobacillus rhamnosus* GG	In vivo (in mice)	Enhancing LCFAs synthesis, strengthening intestinal barrier function, and reducing endotoxemia	[74]
*Lactobacillus rhamnosus* R0011 and *acidophilus* R0052	In vivo (in mice)	Reducing the inflammatory mediators, and downregulating TLR4 expression	[75]
*Akkermansia muciniphila*	In vivo (in mice); in vitro (faeces of ALD patients)	Increasing intestinal barrier integrity, mucus thickness and tight-junction, and decreasing gut leakiness	[69]
**Natural products**			
Flaxseed oil	In vivo (in mice)	Reducing inflammatory cytokines and modulating gut dysbiosis	[24]
Saturated long-chain fatty acids	In vivo (in mice)	Promoting commensal *Lactobacillus* growth, maintaining intestinal eubiosis and gut barrier, and reducing ethanol-induced liver injury	[79]
Korea red ginseng and urushiol from *Rhus verniciflua*	In vivo (in mice)	Attenuating ALD by downregulating TLR4 expression	[75]
Lychee (*Litchi chinensis* Sonn.) pulp phenolic extract	In vivo (in mice)	Alleviating intestinal microbiota dysbiosis, restoring intestinal barrier dysfunction, and suppressing liver inflammation	[80]
Green tea extract	In vivo (in rats)	Preventing non-alcoholic steatohepatitis (NASH) by enhancing the tight junction proteins, decreasing endotoxin leak, and suppressing TLR4/MyD88/NF-κB activation.	[81]
Rhubarb extract	In vivo (in mice)	Protecting the liver from inflammation and oxidative stress partially due to the modulation of the gut microbiota	[82]
Milk osteopontin	In vivo (in mice)	Maintaining intestinal integrity and permeability, normalizing biomarkers and cytokines regarding inflammation, and improving endotoxemia	[83]
Aplysin	In vivo (in rats)	Normalizing the increased plasma endotoxin diamine oxidase (DAO), fatty acid-binding protein 2 (FABP2), and gut microbial composition	[85]
Epigallocatechin gallate (EGCG)	In vivo (in rats)	Showing a prebiotic effect for *L. plantarum*, and decreasing inflammation, necrosis, and apoptosis	[26]
*L. plantarum* (in a double layered microencapsulation)	In vivo (in rats)	Attenuating endotoxemia, normalizing liver biomarkers, reducing NF-κB and cytokines, and restoring the morphology and function of gut and liver	[27]
Indole-3-carbinol	In vivo (in mice)	Reducing oxidative stress and inflammation related to the gut-liver-adipose tissue axis; improving gut integrity and endotoxemia; restoring the antioxidant capacity of the liver and blocking the release of free fatty acids.	[84]

**Table 3:** Effects and mechanisms of natural products and probiotics on liver fibrosis, cirrhosis and cancer by modulating gut microbiota.

Factors that Affect Liver Fibrosis/Cirrhosis/Cancer	Liver Diseases	Study Type	Effects and Mechanism	References
**Probiotics**				
*Saccharomyces boulardii*	CCl_4_-induced liver fibrosis	In vivo (in rats)	Improving gut permeability, normalizing increased serum endotoxin and pro-inflammatory cytokine levels, and modulating microbial composition in gut	[92]
Probiotic beverage Yakult 400 containing *Lactobacillus casei* strain Shirota	Alcoholic liver cirrhosis	In vivo (in human)	Normalizing the gut flora by increasing obligate anerobic bacteria and decreasing Enterobacteriaceae, and improving liver function by increasing serum rapid-turnover protein production	[97]
Probiotic VSL#3	Liver cirrhosis with hepatic encephalopathy (HE)	In vivo (in human)	Reducing the severity of liver cirrhosis and hospitalization	[103]
Probiotic VSL#3 (containing eight, live lyophilized bacterial strains: *Bifidobacterium breve*, *Bifidobacterium longum* (*lactis*), *Bifidobacterium infantis* (*lactis*), *Lactobacillus acidophilus*, *Lactobacillus plantarum*, *Lactobacillus paracasei*, *Lactobacillus bulgaricus* and *Streptococcus thermophilus*)	Liver cirrhosis	In vivo (in human)	Modulating several molecules and compounds, such as MIP-3α/CCL20, NO, TXB2 and MPO	[104]
*Lactobacillus salivarius* LI01 or *Pediococcus pentosaceus* LI05	CCl_4_-induced liver fibrosis	In vivo (in rats)	Improving the disrupted intestinal barrier, downregulating hepatic profibrogenic genes, and inhibiting inflammation	[105]
**Natural products**				
Artesunate	Liver cirrhosis induced by CCl_4_, ethanol, and a high-fat diet (HFD)	In vivo (in rats)	Improving gut microbial dysbiosis, suppressing inflammation, enhancing intestinal mucosal barrier, and reducing bacterial translocation	[106]
Inulin-type fructans	HCC	In vivo (in mice); In vitro (in BaF3 cells)	Reducing hepatic BaF3 cell infiltration, attenuating inflammation, and increasing portal propionate concentration; suppressing BaF3 cell proliferation, and inhibiting BaF3 cell growth cAMP-dependently (propionate)	[107]

## References

[1] WHO Hepatitis—WHO Calls for Better Monitoring of Viral Hepatitis and Liver Cancer. http://www.who.int/hepatitis/news-events/hepatitis-surveillance-protocol-story/en/.

[2] WHO Cancer. http://www.who.int/mediacentre/factsheets/fs297/en/.

[3] WHO Alcohol. http://www.who.int/mediacentre/factsheets/fs349/en/.

[4] Guarner F., Malagelada J.R. (2003). Gut flora in health and disease. Lancet.

[5] Turnbaugh P.J., Ley R.E., Hamady M., Fraser-Liggett C.M., Knight R., Gordon J.I. (2007). The human microbiome project. Nature.

[6] Le Bot N. (2013). Obesity-associated gut microbiota induce liver cancer. Nat. Cell Biol..

[7] Qin N., Yang F., Li A., Prifti E., Chen Y., Shao L., Guo J., Le Chatelier E., Yao J., Wu L. (2014). Alterations of the human gut microbiome in liver cirrhosis. Nature.

[8] Raman M., Ahmed I., Gillevet P.M., Probert C.S., Ratcliffe N.M., Smith S., Greenwood R., Sikaroodi M., Lam V., Crotty P. (2013). Fecal microbiome and volatile organic compound metabolome in obese humans with nonalcoholic fatty liver disease. Clin. Gastroenterol. Hepatol..

[9] Adolph T.E., Grander C., Moschen A.R., Tilg H. (2018). Liver-microbiome axis in health and disease. Trends Immunol..

[10] Stanislawski M.A., Lozupone C.A., Wagner B.D., Eggesbo M., Sontag M.K., Nusbacher N.M., Martinez M., Dabelea D. (2018). Gut microbiota in adolescents and the association with fatty liver: The EPOCH study. Pediatr. Res..

[11] Szabo G. (2015). Gut-liver axis in alcoholic liver disease. Gastroenterology.

[12] Yip L.Y., Aw C.C., Lee S.H., Hong Y.S., Ku H.C., Xu W.H., Chan J., Cheong E., Chng K.R., Ng A. (2018). The liver-gut microbiota axis modulates hepatotoxicity of tacrine in the rat. Hepatology.

[13] Zhang Y.J., Li S., Gan R.Y., Zhou T., Xu D.P., Li H.B. (2015). Impacts of gut bacteria on human health and diseases. Int. J. Mol. Sci..

[14] Krishnan S., Ding Y., Saedi N., Choi M., Sridharan G.V., Sherr D.H., Yarmush M.L., Alaniz R.C., Jayaraman A., Lee K. (2018). Gut microbiota-derived tryptophan metabolites modulate inflammatory response in hepatocytes and macrophages. Cell Rep..

[15] Massey V.L., Stocke K.S., Schmidt R.H., Tan M., Ajami N., Neal R.E., Petrosino J.F., Barve S., Arteel G.E. (2015). Oligofructose protects against arsenic-induced liver injury in a model of environment/obesity interaction. Toxicol. Appl. Pharmacol..

[16] Porras D., Nistal E., Martinez-Florez S., Pisonero-Vaquero S., Olcoz J.L., Jover R., Gonzalez-Gallego J., Garcia-Mediavilla M.V., Sanchez-Campos S. (2017). Protective effect of quercetin on high-fat diet-induced non-alcoholic fatty liver disease in mice is mediated by modulating intestinal microbiota imbalance and related gut-liver axis activation. Free Radic. Biol. Med..

[17] Gan R.Y., Li H.B., Sui Z.Q., Corke H. (2018). Absorption, metabolism, anti-cancer effect and molecular targets of epigallocatechin gallate (EGCG): An updated review. Crit. Rev. Food Sci. Nutr..

[18] Meng X., Li Y., Li S., Gan R.Y., Li H.B. (2018). Natural products for prevention and treatment of chemical-induced liver injuries. Compr. Rev. Food Sci..

[19] Zhang J.J., Meng X., Li Y., Zhou Y., Xu D.P., Li S., Li H.B. (2017). Effects of melatonin on liver injuries and diseases. Int. J. Mol. Sci..

[20] Zhou T., Zhang Y.J., Xu D.P., Wang F., Zhou Y., Zheng J., Li Y., Zhang J.J., Li H.B. (2017). Protective effects of lemon juice on alcohol-induced liver injury in mice. Biomed. Res. Int..

[21] Sekita A., Okazaki Y., Katayama T. (2016). Dietary phytic acid prevents fatty liver by reducing expression of hepatic lipogenic enzymes and modulates gut microflora in rats fed a high-sucrose diet. Nutrition.

[22] Song H., Chu Q., Yan F., Yang Y., Han W., Zheng X. (2016). Red pitaya betacyanins protects from diet-induced obesity, liver steatosis and insulin resistance in association with modulation of gut microbiota in mice. J. Gastroenterol. Hepatol..

[23] Feng Q., Liu W., Baker S.S., Li H., Chen C., Liu Q., Tang S., Guan L., Tsompana M., Kozielski R. (2017). Multi-targeting therapeutic mechanisms of the Chinese herbal medicine QHD in the treatment of non-alcoholic fatty liver disease. Oncotarget.

[24] Zhang X., Wang H., Yin P., Fan H., Sun L., Liu Y. (2017). Flaxseed oil ameliorates alcoholic liver disease via anti-inflammation and modulating gut microbiota in mice. Lipids Health Dis..

[25] Zhao C., Yang C., Chen M., Lv X., Liu B., Yi L., Cornara L., Wei M.C., Yang Y.C., Tundis R. (2018). Regulatory efficacy of brown seaweed *Lessonia nigrescens* extract on the gene expression profile and intestinal microflora in type 2 diabetic mice. Mol. Nutr. Food Res..

[26] Rishi P., Arora S., Kaur U.J., Chopra K., Kaur I.P. (2017). Better management of alcohol liver disease using a ‘Microstructured Synbox’ System comprising *L. plantarum* and EGCG. PLoS One.

[27] Arora S., Kaur I.P., Chopra K., Rishi P. (2014). Efficiency of double layered microencapsulated probiotic to modulate proinflammatory molecular markers for the management of alcoholic liver disease. Mediat. Inflamm..

[28] Lazo M., Hernaez R., Eberhardt M.S., Bonekamp S., Kamel I., Guallar E., Koteish A., Brancati F.L., Clark J.M. (2013). Prevalence of nonalcoholic fatty liver disease in the United States: The Third National Health and Nutrition Examination Survey, 1988-1994. Am. J. Epidemiol..

[29] Ertle J., Dechene A., Sowa J.P., Penndorf V., Herzer K., Kaiser G., Schlaak J.F., Gerken G., Syn W.K., Canbay A. (2011). Non-alcoholic fatty liver disease progresses to hepatocellular carcinoma in the absence of apparent cirrhosis. Int. J. Cancer.

[30] Yang J.D., Kim B., Sanderson S.O., St. Sauver J.L., Yawn B.P., Pedersen R.A., Larson J.J., Therneau T.M., Roberts L.R., Kim W.R. (2012). Hepatocellular carcinoma in Olmsted county, Minnesota, 1976–2008. Mayo Clin. Proc..

[31] Li Y., Zhang J.J., Xu D.P., Zhou T., Zhou Y., Li S., Li H.B. (2016). Bioactivities and health benefits of wild fruits. Int. J. Mol. Sci..

[32] Meng X., Li Y., Li S., Zhou Y., Gan R.Y., Xu D.P., Li H.B. (2017). Dietary sources and bioactivities of melatonin. Nutrients.

[33] Zhang J.J., Li Y., Zhou T., Xu D.P., Zhang P., Li S., Li H.B. (2016). Bioactivities and health benefits of mushrooms mainly from China. Molecules.

[34] Delarue J., Lalles J.P. (2016). Nonalcoholic fatty liver disease: Roles of the gut and the liver and metabolic modulation by some dietary factors and especially long-chain *n*-3 PUFA. Mol. Nutr. Food Res..

[35] Puri P., Sanyal A.J. (2018). The intestinal microbiome in nonalcoholic fatty liver disease. Clin. Liver Dis..

[36] Bibbo S., Ianiro G., Dore M.P. (2018). Gut microbiota as a driver of inflammation in nonalcoholic fatty liver disease. Mediat. Inflamm..

[37] Le Roy T., Llopis M., Lepage P., Bruneau A., Rabot S., Bevilacqua C., Martin P., Philippe C., Walker F., Bado A. (2013). Intestinal microbiota determines development of non-alcoholic fatty liver disease in mice. Gut.

[38] Nobili V., Putignani L., Mosca A., Chierico F.D., Vernocchi P., Alisi A., Stronati L., Cucchiara S., Toscano M., Drago L. (2018). *Bifidobacteria* and *Lactobacilli* in the gut microbiome of children with non-alcoholic fatty liver disease: Which strains act as health players?. Arch. Med. Sci..

[39] Del Chierico F., Nobili V., Vernocchi P., Russo A., Stefanis C., Gnani D., Furlanello C., Zandona A., Paci P., Capuani G. (2017). Gut microbiota profiling of pediatric nonalcoholic fatty liver disease and obese patients unveiled by an integrated meta-omics-based approach. Hepatology.

[40] Borrelli A., Bonelli P., Tuccillo F.M., Goldfine I.D., Evans J.L., Buonaguro F.M., Mancini A. (2018). Role of gut microbiota and oxidative stress in the progression of non-alcoholic fatty liver disease to hepatocarcinoma: Current and innovative therapeutic approaches. Redox. Biol..

[41] Zeng H., Liu J., Jackson M.I., Zhao F.Q., Yan L., Combs G.J. (2013). Fatty liver accompanies an increase in *Lactobacillus* species in the hind gut of C57BL/6 mice fed a high-fat diet. J. Nutr..

[42] De Minicis S., Rychlicki C., Agostinelli L., Saccomanno S., Candelaresi C., Trozzi L., Mingarelli E., Facinelli B., Magi G., Palmieri C. (2014). Dysbiosis contributes to fibrogenesis in the course of chronic liver injury in mice. Hepatology.

[43] Matsushita N., Osaka T., Haruta I., Ueshiba H., Yanagisawa N., Omori-Miyake M., Hashimoto E., Shibata N., Tokushige K., Saito K. (2016). Effect of lipopolysaccharide on the progression of non-alcoholic fatty liver disease in high caloric diet-fed mice. Scand. J. Immunol..

[44] Mahana D., Trent C.M., Kurtz Z.D., Bokulich N.A., Battaglia T., Chung J., Muller C.L., Li H., Bonneau R.A., Blaser M.J. (2016). Antibiotic perturbation of the murine gut microbiome enhances the adiposity, insulin resistance, and liver disease associated with high-fat diet. Genome Med..

[45] Xie G., Wang X., Liu P., Wei R., Chen W., Rajani C., Hernandez B.Y., Alegado R., Dong B., Li D. (2016). Distinctly altered gut microbiota in the progression of liver disease. Oncotarget.

[46] Chiu C.C., Ching Y.H., Li Y.P., Liu J.Y., Huang Y.T., Huang Y.W., Yang S.S., Huang W.C., Chuang H.L. (2017). Nonalcoholic fatty liver disease is exacerbated in high-fat diet-fed gnotobiotic mice by colonization with the gut microbiota from patients with nonalcoholic steatohepatitis. Nutrients.

[47] Boursier J., Mueller O., Barret M., Machado M., Fizanne L., Araujo-Perez F., Guy C.D., Seed P.C., Rawls J.F., David L.A. (2016). The severity of nonalcoholic fatty liver disease is associated with gut dysbiosis and shift in the metabolic function of the gut microbiota. Hepatology.

[48] Fialho A., Fialho A., Thota P., McCullough A.J., Shen B. (2016). Small intestinal bacterial overgrowth is associated with non-alcoholic fatty liver disease. J. Gastrointest. Liver Dis..

[49] Jiang W., Wu N., Wang X., Chi Y., Zhang Y., Qiu X., Hu Y., Li J., Liu Y. (2015). Dysbiosis gut microbiota associated with inflammation and impaired mucosal immune function in intestine of humans with non-alcoholic fatty liver disease. Sci. Rep..

[50] Saltzman E.T., Palacios T., Thomsen M., Vitetta L. (2018). Intestinal microbiome shifts, dysbiosis, inflammation, and non-alcoholic fatty liver disease. Front. Microbiol..

[51] Reid D.T., McDonald B., Khalid T., Vo T., Schenck L.P., Surette M.G., Beck P.L., Reimer R.A., Probert C.S., Rioux K.P. (2016). Unique microbial-derived volatile organic compounds in portal venous circulation in murine non-alcoholic fatty liver disease. Biochim. Biophys. Acta.

[52] Michail S., Lin M., Frey M.R., Fanter R., Paliy O., Hilbush B., Reo N.V. (2015). Altered gut microbial energy and metabolism in children with non-alcoholic fatty liver disease. FEMS Microbiol. Ecol..

[53] Janssen A., Houben T., Katiraei S., Dijk W., Boutens L., van der Bolt N., Wang Z., Brown J.M., Hazen S.L., Mandard S. (2017). Modulation of the gut microbiota impacts nonalcoholic fatty liver disease: A potential role for bile acids. J. Lipid Res..

[54] Yamada S., Takashina Y., Watanabe M., Nagamine R., Saito Y., Kamada N., Saito H. (2018). Bile acid metabolism regulated by the gut microbiota promotes non-alcoholic steatohepatitis-associated hepatocellular carcinoma in mice. Oncotarget.

[55] Xin J., Zeng D., Wang H., Ni X., Yi D., Pan K., Jing B. (2014). Preventing non-alcoholic fatty liver disease through *Lactobacillus johnsonii* BS15 by attenuating inflammation and mitochondrial injury and improving gut environment in obese mice. Appl. Microbiol. Biotechnol..

[56] Ritze Y., Bardos G., Claus A., Ehrmann V., Bergheim I., Schwiertz A., Bischoff S.C. (2014). *Lactobacillus rhamnosus* GG protects against non-alcoholic fatty liver disease in mice. PLoS ONE.

[57] Xue L., He J., Gao N., Lu X., Li M., Wu X., Liu Z., Jin Y., Liu J., Xu J. (2017). Probiotics may delay the progression of nonalcoholic fatty liver disease by restoring the gut microbiota structure and improving intestinal endotoxemia. Sci. Rep..

[58] Rivero-Gutierrez B., Gamez-Belmonte R., Suarez M.D., Lavin J.L., Aransay A.M., Olivares M., Martinez-Augustin O., Sanchez de Medina F., Zarzuelo A. (2017). A synbiotic composed of *Lactobacillus fermentum* CECT5716 and FOS prevents the development of fatty acid liver and glycemic alterations in rats fed a high fructose diet associated with changes in the microbiota. Mol. Nutr. Food Res..

[59] Anhe F.F., Varin T.V., Le Barz M., Pilon G., Dudonne S., Trottier J., St-Pierre P., Harris C.S., Lucas M., Lemire M. (2018). Arctic berry extracts target the gut-liver axis to alleviate metabolic endotoxaemia, insulin resistance and hepatic steatosis in diet-induced obese mice. Diabetologia.

[60] Tian Y., Wang H., Yuan F., Li N., Huang Q., He L., Wang L., Liu Z. (2016). Perilla oil has similar protective effects of fish oil on high-fat diet-induced nonalcoholic fatty liver disease and gut dysbiosis. Biomed. Res. Int..

[61] Jegatheesan P., Beutheu S., Freese K., Waligora-Dupriet A.J., Nubret E., Butel M.J., Bergheim I., De Bandt J.P. (2016). Preventive effects of citrulline on western diet-induced non-alcoholic fatty liver disease in rats. Br. J. Nutr..

[62] Jegatheesan P., Beutheu S., Ventura G., Sarfati G., Nubret E., Kapel N., Waligora-Dupriet A.J., Bergheim I., Cynober L., De-Bandt J.P. (2016). Effect of specific amino acids on hepatic lipid metabolism in fructose-induced non-alcoholic fatty liver disease. Clin. Nutr..

[63] Lin P., Lu J., Wang Y., Gu W., Yu J., Zhao R. (2015). Naturally occurring stilbenoid TSG reverses non-alcoholic fatty liver diseases via gut-liver axis. PLoS ONE.

[64] Everard A., Belzer C., Geurts L., Ouwerkerk J.P., Druart C., Bindels L.B., Guiot Y., Derrien M., Muccioli G.G., Delzenne N.M. (2013). Cross-talk between *Akkermansia muciniphila* and intestinal epithelium controls diet-induced obesity. Proc. Natl. Acad. Sci. USA.

[65] Zhou Y., Zheng J., Li S., Zhou T., Zhang P., Li H.B. (2016). Alcoholic beverage consumption and chronic diseases. Int. J. Environ. Res. Public Health.

[66] Arsene D., Farooq O., Bataller R. (2016). New therapeutic targets in alcoholic hepatitis. Hepatol. Int..

[67] Wang F., Li Y., Zhang Y.J., Zhou Y., Li S., Li H.B. (2016). Natural products for the prevention and treatment of hangover and alcohol use disorder. Molecules.

[68] Wang F., Zhang Y.J., Zhou Y., Li Y., Zhou T., Zheng J., Zhang J.J., Li S., Xu D.P., Li H.B. (2016). Effects of beverages on alcohol metabolism: Potential health benefits and harmful impacts. Int. J. Mol. Sci..

[69] Grander C., Adolph T.E., Wieser V., Lowe P., Wrzosek L., Gyongyosi B., Ward D.V., Grabherr F., Gerner R.R., Pfister A. (2017). Recovery of ethanol-induced *Akkermansia muciniphila* depletion ameliorates alcoholic liver disease. Gut.

[70] Cho Y.E., Yu L.R., Abdelmegeed M.A., Yoo S.H., Song B.J. (2018). Apoptosis of enterocytes and nitration of junctional complex proteins promote alcohol-induced gut leakiness and liver injury. J. Hepatol..

[71] Chang B., Sang L., Wang Y., Tong J., Wang B. (2013). The role of FoxO4 in the relationship between alcohol-induced intestinal barrier dysfunction and liver injury. Int. J. Mol. Med..

[72] Llopis M., Cassard A.M., Wrzosek L., Boschat L., Bruneau A., Ferrere G., Puchois V., Martin J.C., Lepage P., Le Roy T. (2016). Intestinal microbiota contributes to individual susceptibility to alcoholic liver disease. Gut.

[73] Wang Y., Liu Y., Kirpich I., Ma Z., Wang C., Zhang M., Suttles J., McClain C., Feng W. (2013). *Lactobacillus rhamnosus* GG reduces hepatic TNFα production and inflammation in chronic alcohol-induced liver injury. J. Nutr. Biochem..

[74] Shi X., Wei X., Yin X., Wang Y., Zhang M., Zhao C., Zhao H., McClain C.J., Feng W., Zhang X. (2015). Hepatic and fecal metabolomic analysis of the effects of *Lactobacillus rhamnosus* GG on alcoholic fatty liver disease in mice. J. Proteome Res..

[75] Hong M., Kim S.W., Han S.H., Kim D.J., Suk K.T., Kim Y.S., Kim M.J., Kim M.Y., Baik S.K., Ham Y.L. (2015). Probiotics (*Lactobacillus rhamnosus* R0011 and *acidophilus* R0052) reduce the expression of toll-like receptor 4 in mice with alcoholic liver disease. PLoS ONE.

[76] Li S., Gan L.Q., Li S.K., Zheng J.C., Xu D.P., Li H.B. (2014). Effects of herbal infusions, tea and carbonated beverages on alcohol dehydrogenase and aldehyde dehydrogenase activity. Food Funct..

[77] Zhang Y.J., Wang F., Zhou Y., Li Y., Zhou T., Zheng J., Zhang J.J., Li S., Xu D.P., Li H.B. (2016). Effects of 20 selected fruits on ethanol metabolism: Potential health benefits and harmful impacts. Int. J. Environ. Res. Public Health.

[78] Zhang Y.J., Zhou T., Wang F., Zhou Y., Li Y., Zhang J.J., Zheng J., Xu D.P., Li H.B. (2016). The effects of Syzygium samarangense, Passiflora edulis and Solanum muricatum on alcohol-induced liver injury. Int. J. Mol. Sci..

[79] Chen P., Torralba M., Tan J., Embree M., Zengler K., Starkel P., van Pijkeren J.P., DePew J., Loomba R., Ho S.B. (2015). Supplementation of saturated long-chain fatty acids maintains intestinal eubiosis and reduces ethanol-induced liver injury in mice. Gastroenterology.

[80] Xiao J., Zhang R., Zhou Q., Liu L., Huang F., Deng Y., Ma Y., Wei Z., Tang X., Zhang M. (2017). Lychee (*Litchi chinensis* Sonn.) pulp phenolic extract provides protection against alcoholic liver injury in mice by alleviating intestinal microbiota dysbiosis, intestinal barrier dysfunction, and liver inflammation. J. Agric. Food Chem..

[81] Chung M.Y., Mah E., Masterjohn C., Noh S.K., Park H.J., Clark R.M., Park Y.K., Lee J.Y., Bruno R.S. (2015). Green tea lowers hepatic COX-2 and prostaglandin E_2_ in rats with dietary fat-induced nonalcoholic steatohepatitis. J. Med. Food.

[82] Neyrinck A.M., Etxeberria U., Taminiau B., Daube G., Van Hul M., Everard A., Cani P.D., Bindels L.B., Delzenne N.M. (2017). Rhubarb extract prevents hepatic inflammation induced by acute alcohol intake, an effect related to the modulation of the gut microbiota. Mol. Nutr. Food Res..

[83] Ge X., Lu Y., Leung T.M., Sorensen E.S., Nieto N. (2013). Milk osteopontin, a nutritional approach to prevent alcohol-induced liver injury. Am. J. Physiol. Gastrointest. Liver Physiol..

[84] Choi Y., Abdelmegeed M.A., Song B.J. (2018). Preventive effects of indole-3-carbinol against alcohol-induced liver injury in mice via antioxidant, anti-inflammatory, and anti-apoptotic mechanisms: Role of gut-liver-adipose tissue axis. J. Nutr. Biochem..

[85] Xue M., Liu Y., Lyu R., Ge N., Liu M., Ma Y., Liang H. (2017). Protective effect of aplysin on liver tissue and the gut microbiota in alcohol-fed rats. PLoS ONE.

[86] Teixeira R., Marcos L.A., Friedman S.L. (2007). Immunopathogenesis of hepatitis C virus infection and hepatic fibrosis: New insights into antifibrotic therapy in chronic hepatitis C. Hepatol. Res..

[87] Frasinariu O.E., Ceccarelli S., Alisi A., Moraru E., Nobili V. (2013). Gut-liver axis and fibrosis in nonalcoholic fatty liver disease: An input for novel therapies. Dig. Liver Dis..

[88] Hartmann P., Haimerl M., Mazagova M., Brenner D.A., Schnabl B. (2012). Toll-like receptor 2-mediated intestinal injury and enteric tumor necrosis factor receptor I contribute to liver fibrosis in mice. Gastroenterology.

[89] Kuntzen C., Schwabe R.F. (2017). Gut microbiota and Toll-like receptors set the stage for cytokine-mediated failure of antibacterial responses in the fibrotic liver. Gut.

[90] Hackstein C.P., Assmus L.M., Welz M., Klein S., Schwandt T., Schultze J., Forster I., Gondorf F., Beyer M., Kroy D. (2017). Gut microbial translocation corrupts myeloid cell function to control bacterial infection during liver cirrhosis. Gut.

[91] Aoyama T., Kuwahara-Arai K., Uchiyama A., Kon K., Okubo H., Yamashina S., Ikejima K., Kokubu S., Miyazaki A., Watanabe S. (2017). Spleen-derived lipocalin-2 in the portal vein regulates Kupffer cells activation and attenuates the development of liver fibrosis in mice. Lab. Investig..

[92] Li M., Zhu L., Xie A., Yuan J. (2015). Oral administration of *Saccharomyces boulardii* ameliorates carbon tetrachloride-induced liver fibrosis in rats via reducing intestinal permeability and modulating gut microbial composition. Inflammation.

[93] Liu Y., Jin Y., Li J., Zhao L., Li Z., Xu J., Zhao F., Feng J., Chen H., Fang C. (2018). Small bowel transit and altered gut microbiota in patients with liver cirrhosis. Front. Physiol..

[94] Liu J., Wu D., Ahmed A., Li X., Ma Y., Tang L., Mo D., Ma Y., Xin Y. (2012). Comparison of the gut microbe profiles and numbers between patients with liver cirrhosis and healthy individuals. Curr. Microbiol..

[95] Wu Z.W., Ling Z.X., Lu H.F., Zuo J., Sheng J.F., Zheng S.S., Li L.J. (2012). Changes of gut bacteria and immune parameters in liver transplant recipients. Hepatob. Pancreat. Dis. Int..

[96] Grat M., Holowko W., Galecka M., Grat K., Szachtaz P., Lewandowsk Z., Kosinska I., Schmidts M., Olejnik-Schmidt A., Krawczyk M. (2014). Gut microbiota in cirrhotic liver transplant candidates. Hepatogastroenterology.

[97] Koga H., Tamiya Y., Mitsuyama K., Ishibashi M., Matsumoto S., Imaoka A., Hara T., Nakano M., Ooeda K., Umezaki Y. (2013). Probiotics promote rapid-turnover protein production by restoring gut flora in patients with alcoholic liver cirrhosis. Hepatol. Int..

[98] Tuomisto S., Pessi T., Collin P., Vuento R., Aittoniemi J., Karhunen P.J. (2014). Changes in gut bacterial populations and their translocation into liver and ascites in alcoholic liver cirrhotics. BMC Gastroenterol..

[99] Dubinkina V.B., Tyakht A.V., Odintsova V.Y., Yarygin K.S., Kovarsky B.A., Pavlenko A.V., Ischenko D.S., Popenko A.S., Alexeev D.G., Taraskina A.Y. (2017). Links of gut microbiota composition with alcohol dependence syndrome and alcoholic liver disease. Microbiome.

[100] Ahluwalia V., Betrapally N.S., Hylemon P.B., White M.B., Gillevet P.M., Unser A.B., Fagan A., Daita K., Heuman D.M., Zhou H. (2016). Impaired gut-liver-brain axis in patients with cirrhosis. Sci. Rep..

[101] Assimakopoulos S.F., Tsamandas A.C., Tsiaoussis G.I., Karatza E., Zisimopoulos D., Maroulis I., Kontogeorgou E., Georgiou C.D., Scopa C.D., Thomopoulos K.C. (2013). Intestinal mucosal proliferation, apoptosis and oxidative stress in patients with liver cirrhosis. Ann. Hepatol..

[102] Ahluwalia V., Wade J.B., Heuman D.M., Hammeke T.A., Sanyal A.J., Sterling R.K., Stravitz R.T., Luketic V., Siddiqui M.S., Puri P. (2014). Enhancement of functional connectivity, working memory and inhibitory control on multi-modal brain MR imaging with rifaximin in cirrhosis: Implications for the gut-liver-brain axis. Metab. Brain Dis..

[103] Dhiman R.K., Rana B., Agrawal S., Garg A., Chopra M., Thumburu K.K., Khattri A., Malhotra S., Duseja A., Chawla Y.K. (2014). Probiotic VSL#3 reduces liver disease severity and hospitalization in patients with cirrhosis: A randomized, controlled trial. Gastroenterology.

[104] Marlicz W., Wunsch E., Mydlowska M., Milkiewicz M., Serwin K., Mularczyk M., Milkiewicz P., Raszeja-Wyszomirska J. (2016). The effect of short term treatment with probiotic VSL#3 on various clinical and biochemical parameters in patients with liver cirrhosis. J. Physiol. Pharmacol..

[105] Shi D., Lv L., Fang D., Wu W., Hu C., Xu L., Chen Y., Guo J., Hu X., Li A. (2017). Administration of Lactobacillus salivarius LI01 or Pediococcus pentosaceus LI05 prevents CCl_4_-induced liver cirrhosis by protecting the intestinal barrier in rats. Sci. Rep..

[106] Chen Y.X., Lai L.N., Zhang H.Y., Bi Y.H., Meng L., Li X.J., Tian X.X., Wang L.M., Fan Y.M., Zhao Z.F. (2016). Effect of artesunate supplementation on bacterial translocation and dysbiosis of gut microbiota in rats with liver cirrhosis. World J. Gastroenterol..

[107] Bindels L.B., Porporato P., Dewulf E.M., Verrax J., Neyrinck A.M., Martin J.C., Scott K.P., Buc C.P., Feron O., Muccioli G.G. (2012). Gut microbiota-derived propionate reduces cancer cell proliferation in the liver. Br. J. Cancer.

[108] WHO World Cancer Report—WHO. http://publications.iarc.fr/Non-Series-Publications/World-Cancer-Reports/World-Cancer-Report-2014.

[109] Deng G.F., Xu X.R., Zhang Y., Li D., Gan R.Y., Li H.B. (2013). Phenolic compounds and bioactivities of pigmented rice. Crit. Rev. Food Sci. Nutr..

[110] Li F., Li S., Li H.B., Deng G.F., Ling W.H., Xu X.R. (2013). Antiproliferative activities of tea and herbal infusions. Food Funct..

[111] Li A.N., Li S., Zhang Y.J., Xu X.R., Chen Y.M., Li H.B. (2014). Resources and biological activities of natural polyphenols. Nutrients.

[112] Zheng J., Zhou Y., Li Y., Xu D.P., Li S., Li H.B. (2016). Spices for prevention and treatment of cancers. Nutrients.

[113] Zhou Y., Li Y., Zhou T., Zheng J., Li S., Li H.B. (2016). Dietary natural products for prevention and treatment of liver cancer. Nutrients.

[114] Li Y., Li S., Zhou Y., Meng X., Zhang J.J., Xu D.P., Li H.B. (2017). Melatonin for the prevention and treatment of cancer. Oncotarget.

[115] Huang R., Li T., Ni J., Bai X., Gao Y., Li Y., Zhang P., Gong Y. (2018). Different sex-based responses of gut microbiota during the development of hepatocellular carcinoma in liver-specific *Tsc1*-Knockout mice. Front. Microbiol..

[116] Grat M., Wronka K.M., Krasnodebski M., Masior L., Lewandowski Z., Kosinska I., Grat K., Stypulkowski J., Rejowski S., Wasilewicz M. (2016). Profile of gut microbiota associated with the presence of hepatocellular cancer in patients with liver cirrhosis. Transplant. Proc..

[117] Yoshimoto S., Loo T.M., Atarashi K., Kanda H., Sato S., Oyadomari S., Iwakura Y., Oshima K., Morita H., Hattori M. (2013). Obesity-induced gut microbial metabolite promotes liver cancer through senescence secretome. Nature.

[118] Loo T.M., Kamachi F., Watanabe Y., Yoshimoto S., Kanda H., Arai Y., Nakajima-Takagi Y., Iwama A., Koga T., Sugimoto Y. (2017). Gut microbiota promotes obesity-associated liver cancer through PGE_2_-mediated suppression of antitumor immunity. Cancer Discov..

[119] Orci L.A., Lacotte S., Delaune V., Slits F., Oldani G., Lazarevic V., Rossetti C., Rubbia-Brandt L., Morel P., Toso C. (2018). Effects of the gut-liver axis on ischaemia-mediated hepatocellular carcinoma recurrence in the mouse liver. J. Hepatol..

[120] Xie G., Wang X., Huang F., Zhao A., Chen W., Yan J., Zhang Y., Lei S., Ge K., Zheng X. (2016). Dysregulated hepatic bile acids collaboratively promote liver carcinogenesis. Int. J. Cancer.

[121] Ma C., Han M., Heinrich B. (2018). Gut microbiome-mediated bile acid metabolism regulates liver cancer via NKT cells. Science.

